# Point Prevalence Survey of Antimicrobial Use in a Tertiary Care Hospital in India Using the WHO Access, Watch, and Reserve (AWaRe) Classification and Its Antimicrobial Stewardship Implications

**DOI:** 10.7759/cureus.108626

**Published:** 2026-05-11

**Authors:** Sujitha Sethuramalingam, Saranya Devi Shanmugam, Velvignesh Sethuramalingam, Srinika Periyasamy, Thrisha Kandakumar

**Affiliations:** 1 Pharmacology, Government Medical College and Hospital Pudukkottai, Pudukkottai, IND; 2 Pharmacology, Madurai Medical College, Madurai, IND

**Keywords:** antimicrobial consumption, antimicrobial prescription, antimicrobial stewardship, hospitalized patients, point prevalence survey, who aware classification

## Abstract

Background: Antimicrobial resistance is largely driven by inappropriate antimicrobial use. The World Health Organization Access, Watch, and Reserve (AWaRe) classification recommends that most of the total antimicrobial consumption should be from the ‘Access’ group, as these agents are associated with a lower risk of resistance. This study aimed to evaluate antimicrobial utilization patterns and AWaRe distribution in a tertiary care hospital in India, and to identify targets for strengthening antimicrobial stewardship.

Methods: A cross-sectional point prevalence survey was conducted in March 2025 among inpatients at a government medical college hospital in Tamil Nadu, India. Data on antimicrobial prescriptions, including indication, number of agents, route of administration, stop date, and culture results, were collected using standardized WHO point prevalence survey forms. Data were analyzed using descriptive statistics and expressed as frequencies and percentages.

Results: A total of 351 patients were surveyed, of whom 192 (54.7%) were receiving antimicrobials. Among 245 antimicrobial prescriptions, 132 (53.9%) were empirical, while 113 (46.1%) were guided by susceptibility testing. Overall, 167 (68.2%) were administered intravenously, and 101 (41.2%) of prescriptions had no documented stop date. Third-generation cephalosporins were the most commonly prescribed agents. 'Watch' group antimicrobials accounted for 159 (64.9%) prescriptions, and 'Access' group accounted for 86 (35.1%) prescriptions, which was substantially below the WHO-recommended target. Notably, no 'Reserve' group antimicrobials were prescribed.

Conclusion: High use of the ‘Watch’ group antimicrobials, inadequate documentation of stop dates, predominant empirical prescribing, and extensive parenteral administration indicate possible gaps in antimicrobial stewardship practices. Strengthening stewardship intervention by increasing ‘Access’ group use, improving prescription documentation, and promoting targeted therapy with timely de-escalation are essential to optimize antimicrobial use in this setting.

## Introduction

Antimicrobial resistance is a global health concern that threatens modern medical practice. The overuse and misuse of antimicrobials in humans, livestock, and agriculture have played a major role in the emergence and spread of antimicrobial resistance. Inappropriate prescriptions, including wrong antimicrobials, incorrect doses, and wrong durations, further worsen the issue. Widespread availability of over-the-counter antimicrobials, use of substandard and counterfeit drugs, self-medication, and poor patient compliance contribute to the development of resistance. Suboptimal rapid diagnostics, inadequate infection control, and environmental contamination also have an impact. Compounding this crisis is the limited discovery of new antimicrobials, with development hampered by scientific, financial, and regulatory barriers. Antimicrobial resistance makes treatment challenging and increases mortality, morbidity, and health care costs [1,2].

According to the WHO 2025 Global Antimicrobial Resistance and Use Surveillance System (GLASS) report, resistance to third-generation cephalosporins, fluoroquinolones, and carbapenems is increasing. One in six bacterial infections is caused by antimicrobial-resistant pathogens [3]. Human deaths due to antimicrobial resistance are currently about five million per year globally, and is predicted to reach 10 million annually by 2050 if no action is taken [4].

In response to this growing threat, the WHO, through the global action plan, has emphasized the need for surveillance of antimicrobial use and stewardship in healthcare settings to combat antimicrobial resistance [5-7]. To promote antimicrobial stewardship and reduce resistance, the WHO developed the Access, Watch, and Reserve (AWaRe) classification of antimicrobials based on their spectrum of activity, clinical importance, and risk of resistance emergence [8].

Our facility currently lacks patient-level data regarding the quantity and quality of antimicrobial prescriptions, which is essential for evaluating the appropriateness of their use. This point prevalence survey aims to establish use patterns, assess prescription quality, and monitor the frequency of antimicrobial utilization according to the AWaRe categories to identify specific targets for enhancing antimicrobial stewardship.

## Materials and methods

This was a cross-sectional point prevalence survey conducted over a week in March 2025 in the wards of the Government Medical College Hospital, Pudukkottai, Tamil Nadu, India. The study was approved by the Institutional Ethics Committee of the Government Medical College & Hospital, Pudukkottai. The wards surveyed were General Medicine, General Surgery, Pediatrics, Obstetrics and Gynecology, Orthopedics, Dermatology, Psychiatry, ENT, Ophthalmology, and Intensive Care Units. The study was conducted as per the National ethical guidelines for biomedical and health research involving human participants (ICMR 2017). As this was a non-interventional study and did not involve any patient examination, interview, or intervention, and since personal identifiers of the patient were not recorded, informed consent of the patient was not required. All the data collected from the case files were kept anonymous and confidential.

Eligibility criteria

All inpatients admitted to the ward on or before 9:00 am on the day of the survey were included.* *Patients admitted to the ward after 9:00 am on the day of the survey, daycare patients, casualty ward patients, and discharged patients waiting for transportation were excluded from the survey. Antifungals, antimalarials, antitubercular drugs, and topical antimicrobials were excluded from the survey.

As per the WHO point prevalence survey methodology, all eligible inpatients present in the selected wards at the time of survey were included; hence, no formal sample size calculation was performed. 

Study procedure and data collection tools

The study was performed according to the WHO point prevalence survey methodology of antimicrobial use, with a modification that included all inpatients admitted to the ward on or before 9:00 am, instead of 8:00 am as per the WHO method, and inclusion of a stop date [9]. The data was extracted from the patient’s case file. The data were collected in two predesigned forms, namely the ward form and the patient form. The ward form contained information such as the date of the survey, name of the ward, number of beds in the ward, total number of inpatients in the ward, and number of patients on oral and parenteral antimicrobials. The patient form captured the details such as the date of admission, gender, age, weight of the patient, diagnosis, systemic antimicrobial information such as the name, dose, frequency, route of administration, and duration of treatment. Antimicrobials used for the treatment of tuberculosis and antivirals were not considered. The following antimicrobial prescribing quality indicators were assessed: documentation of diagnosis, documentation of indication for antimicrobial use, blood culture testing, use of generic names, and documentation of stop date. Consultants were contacted to get the details of missing and incomplete records.

Statistical analysis

The collected data were entered into a Microsoft Excel spreadsheet, and descriptive analysis was performed. Continuous variables such as age and the length of stay in hospital were expressed as median (interquartile range (IQR)), and categorical variables such as sex and antimicrobial quality indicators were expressed in frequencies and percentages. The point prevalence of antimicrobial use was calculated. The most common antimicrobials used in the inpatients of the hospital were categorized according to AWaRe classification 2025 and WHO Anatomical Therapeutic Chemical (ATC) classification [10]. Data were presented as tables and graphs as appropriate. Bar graphs were used to map the consumption of oral and intravenous antimicrobials. Inferential statistical analysis was not performed as the primary objective was to describe antimicrobial utilization patterns at a single point in time.

## Results

A total of 351 patients admitted to the hospital were surveyed for this point prevalence study (Table 1). Of these, 192 (54.7%; 95%CI: 49.5%-59.9%) patients were on antimicrobials. This indicates that more than half of the hospitalized patients were exposed to antimicrobials at a single point in time. Female patients (n=104, 54.2%) were more than male patients (n=88, 45.8%). The characteristics of patients receiving antimicrobials by ward are given in Table 2.

**Table 1:** Baseline characteristics of patients in the wards surveyed IQR: interquartile range

Number of patients	Age (years), median (IQR)	Male, n (%)	Female, n (%)	Length of hospital stay (days), median (IQR)
Characteristics of all inpatients admitted to the wards surveyed (N=351)	38.5 (21.2-55.7)	168 (47.9%)	183 (52.1%)	6 (3-5)
Characteristics of patients on antimicrobials in wards surveyed (N=192)	35.5 (19.7-51.2)	88 (45.8%)	104 (54.2%)	4 (3-4)

**Table 2:** Characteristics of patients receiving antimicrobials by ward IQR: interquartile range

Ward	Age (years), median (IQR)	Gender, n (%)		Length of hospital stay (days), median (IQR)
		Male	Female	
Medicine, n=38	52.5 (45-61)	14 (36.8)	24 (63.2)	3 (3-4)
Surgery, n=30	35.5 (30.2-55)	22 (73.3)	8 (26.7)	4 (2-13)
Obstetrics & Gynecology, n=36	25 (23-27.2)	0 (0)	36 (100)	3 (2-5)
Pediatrics, n=6	6.5 (5.2-7.2)	1(16.7)	5 (83.3)	4 (3.2-5.5)
Orthopedics, n=30	50 (36.5-58.5)	24 (80)	6 (20)	6 (4-8.5)
Intensive care units, n=33	3.5 (2.5-15.2)	17 (51.5)	16 (48.5)	3 (3)
ENT, n=11	21 (13.5-31.5)	5 (45.5)	6 (54.5)	4 (2.5-8)
Ophthalmology, n=6	65 (59-65)	3 (50)	3 (50)	3.5 (3-4)
Dermatology, n=2	76 (74-78)	2 (100)	0 (0)	7.5 (5.7-9.2)
Psychiatry, n=0	0 (0)	0 (0)	0 (0)	0 (0)

The prevalence of antimicrobial use ward-wise is shown in Table 3. Most patients (75%) received a single antimicrobial, while approximately one-fourth received combination therapy (Table 4). Of the 36 patients on antimicrobials in the Obstetrics and Gynecology ward, 29 (80.6%) patients were on two antimicrobials.

**Table 3:** Prevalence of antimicrobial use by ward

Ward	Patients surveyed (N)	Patients on oral/intravenous antimicrobials, n/N (%)	Patients on intravenous antimicrobials, n1/N (%)	Patients on oral antimicrobials, n2/N (%)
Medicine	123	38/123 (30.9)	37/123 (30.1)	4/123 (3.3)
Surgery	44	30/44 (68.1)	22/44 (50)	8/44 (18.2)
Obstetrics & Gynecology	50	36/50 (72)	18/50 (36)	22/50 (44)
Pediatrics	7	6/7 (85.7)	6/7 (85.7)	0 (0)
Orthopedics	50	30/50 (60)	22/50 (44)	11/50 (22)
Intensive care units	50	33/50 (66)	31/50 (62)	5/50 (10)
ENT	12	11/12 (91.7)	11/12 (91.7)	0 (0)
Ophthalmology	11	6/11 (54.5)	0 (0)	6/11 (54.5)
Dermatology	2	2/2 (100)	0 (0)	2/2 (100)
Psychiatry	2	0 (0)	0 (0)	0 (0)
Total	351	192/351 (54.7)	147/351 (41.9)	58/351 (16.5)

**Table 4:** Distribution of patients according to number of antimicrobials prescribed ward wise (N=245)

Ward	Number of patients on antimicrobials	Patients, n (%)		
		Single antimicrobial	Two antimicrobials	>2 antimicrobials
Medicine	38	35 (92.1)	3 (7.9)	0 (0)
Surgery	30	29 (96.7)	0 (0)	1 (3.3)
Obstetrics & Gynecology	36	7 (19.4)	29 (80.6)	0 (0)
Pediatrics	6	6 (100)	0 (0)	0 (0)
Orthopedics	30	25 (83.3)	5 (16.7)	0 (0)
Intensive care units	33	24 (72.7)	7 (21.2)	2 (6.1)
ENT	11	10 (90.9)	0 (0)	1 (9.1)
Ophthalmology	6	6 (100)	0 (0)	0 (0)
Dermatology	2	2 (100)	0 (0)	0 (0)
Psychiatry	0	0 (0)	0 (0)	0 (0)
Total	192	144 (75)	44 (22.9)	4 (2.1)

Analysis of 245 antimicrobial prescriptions from 192 patients found that, despite a predominance of generic prescribing and therapeutic use, most prescriptions were empirical. Further, many prescriptions lacked documentation of stop dates and did not clearly specify the indication for antimicrobial use (Table 5).

**Table 5:** Quality indicators for antimicrobial use (N=245)

Parameter	Category	Frequency (Percentage)
Type of treatment	Empirical	132 (53.9)
	Targeted	113 (46.1)
Purpose of prescription	Therapeutic	202 (82.4)
	Prophylactic	43 (17.6)
Documentation of indication	Documented	184 (75.1)
	Not documented	61 (24.9)
Type of prescription	Generic	230 (93.9)
	Branded	15 (6.1)
Stop date mentioned	Yes	144 (58.8)
	No	101 (41.2)

**Figure 1:**
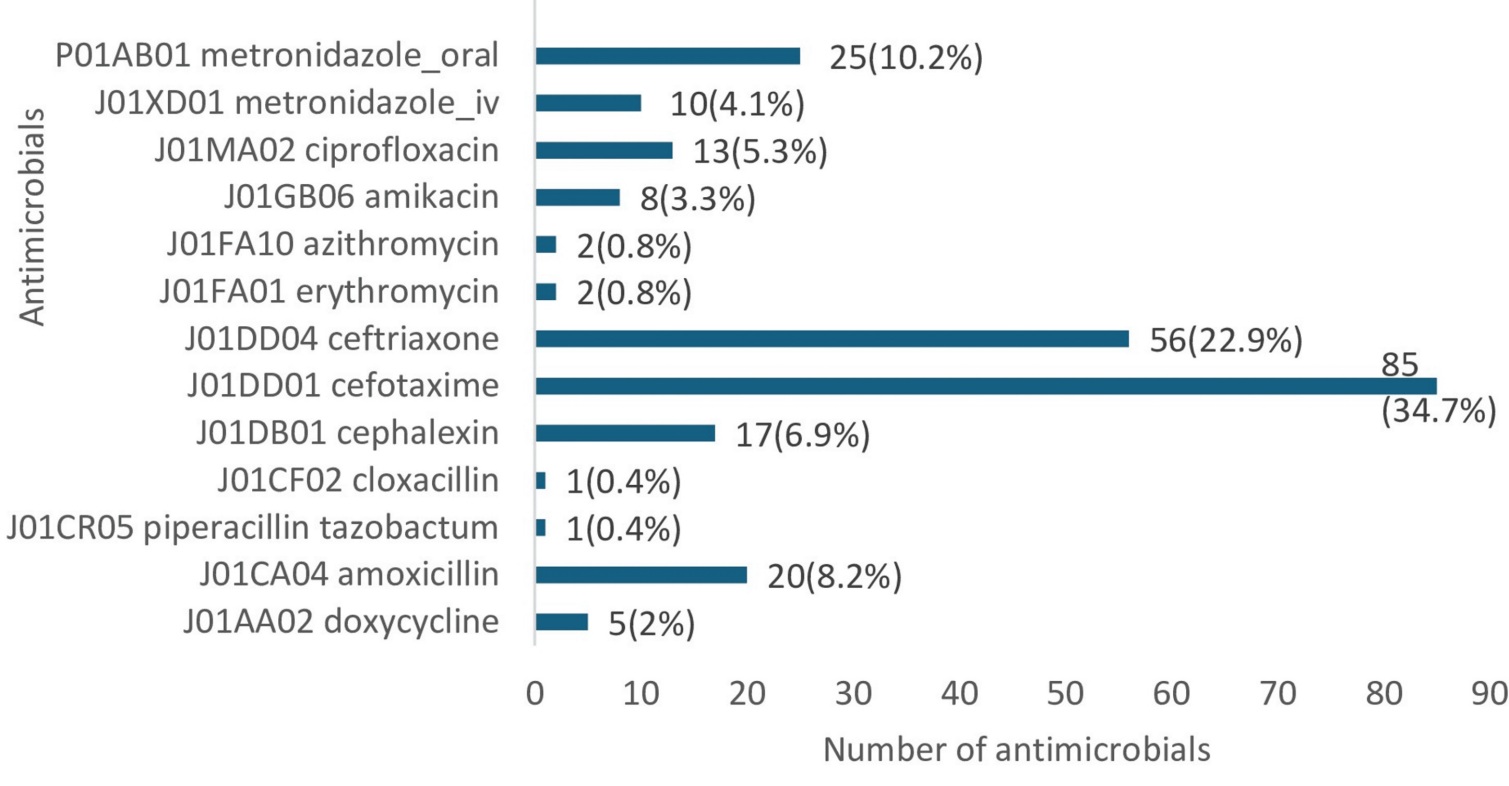
Distribution of antimicrobials (N=245) among inpatients as per the WHO ATC classification (ATC5 codes), showing frequency of individual agents ATC: Anatomical Therapeutic Chemical

Figure 1 shows the prescribed antimicrobials as per the WHO ATC classification system, which indicates that third-generation cephalosporins, cefotaxime, and ceftriaxone were the most prescribed antimicrobials.

**Figure 2:**
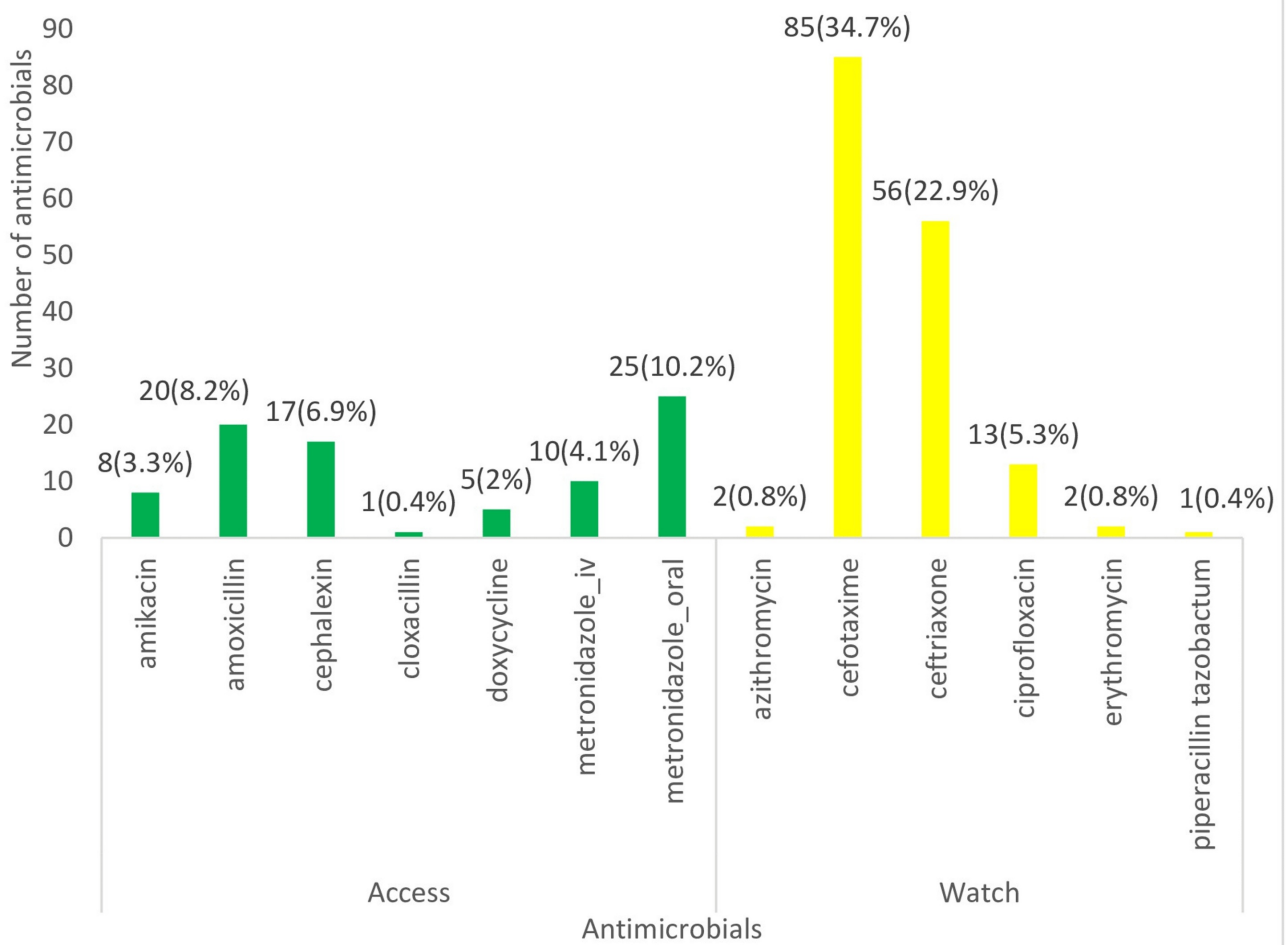
Distribution of antimicrobial agents within Access and Watch categories (N=245)

Upon distribution according to the AWaRe classification system, it was seen that the majority of prescriptions belonged to the 'Watch' group (n=159, 64.9%), exceeding the 'Access' group (n=86, 35.1%). This reflects the predominance of broad-spectrum antimicrobial use in the study setting. As per the consumption of antimicrobials by AWaRe category, oral metronidazole was the most commonly prescribed ‘Access’ category antimicrobial, and cefotaxime was the most commonly prescribed ‘Watch’ category antimicrobial (Figure 2).

**Figure 3:**
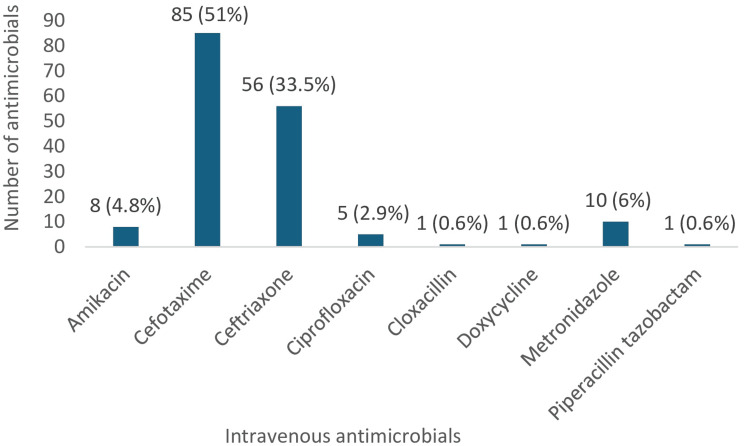
Distribution of intravenous antimicrobial prescriptions (N=167) among inpatients

**Figure 4:**
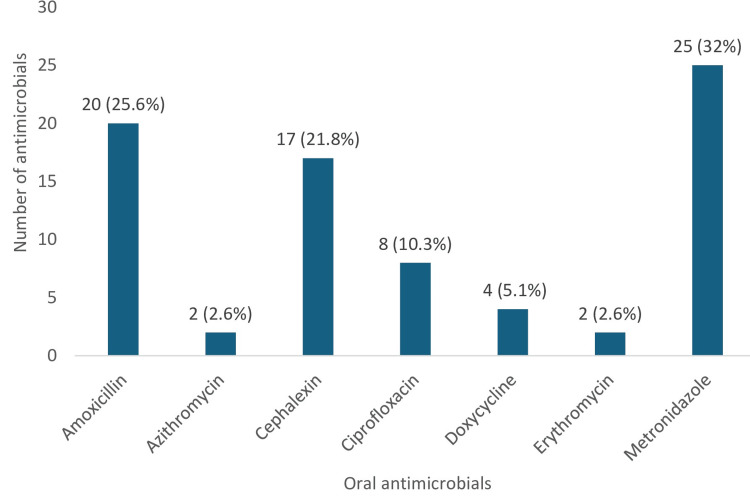
Distribution of oral antimicrobial prescriptions (N=78) among inpatients

All antimicrobial prescriptions were from the WHO Essential Medicines List 2025 [8]. Of the overall antimicrobials, 167 (68.2%) were administered parenterally, and 78 (31.8%) were given orally. This suggests a strong preference for parenteral therapy over oral administration. The ward-wise pattern of parenteral and oral antimicrobial prescriptions is presented in Tables 6, 7. Cefotaxime was the most prescribed parenteral antimicrobial (Figure 3). Metronidazole was the commonly prescribed oral antimicrobial (Figure 4). 

**Table 6:** Intravenous antimicrobial prescription pattern across wards

Ward	Number of parenteral antimicrobial prescriptions	Antimicrobial	Numbers (%)
ENT	14	Amikacin	1 (7.1)
		Cefotaxime	10 (71.6)
		Ceftriaxone	1 (7.1)
		Ciprofloxacin	1 (7.1)
		Metronidazole	1 (7.1)
Intensive care units	38	Amikacin	6 (15.9)
		Cefotaxime	14 (36.8)
		Ceftriaxone	13 (34.2)
		Ciprofloxacin	2 (5.3)
		Cloxacillin	1 (2.6)
		Doxycycline	1 (2.6)
		Metronidazole	1 (2.6)
Medicine	37	Cefotaxime	12 (32.4)
		Ceftriaxone	23 (62.2)
		Ciprofloxacin	2 (5.4)
Obstetrics & Gynecology	25	Cefotaxime	17 (68)
		Metronidazole	7 (28)
		Piperacillin Tazobactam	1 (4)
Orthopedics	23	Cefotaxime	17 (74)
		Ceftriaxone	6 (26)
Pediatrics	6	Cefotaxime	3 (50)
		Ceftriaxone	3 (50)
Surgery	24	Amikacin	1 (4.2)
		Cefotaxime	12 (50)
		Ceftriaxone	10 (41.6)
		Metronidazole	1 (4.2)

**Table 7:** Oral antimicrobial prescription pattern across wards

Ward	Number of oral antimicrobial prescriptions	Antimicrobial	Numbers (%)
Dermatology	2	Cephalexin	1 (50)
		Erythromycin	1 (50)
Intensive care units	6	Amoxicillin	2 (33.2)
		Azithromycin	1 (16.7)
		Doxycycline	1 (16.7)
		Erythromycin	1 (16.7)
		Metronidazole	1 (16.7)
Medicine	4	Doxycycline	3 (75)
		Metronidazole	1 (25)
Obstetrics & Gynecology	40	Amoxicillin	18 (45)
		Metronidazole	22 (55)
Ophthalmology	6	Ciprofloxacin	6 (100)
Orthopedics	12	Cephalexin	10 (83.4)
		Ciprofloxacin	1 (8.3)
		Metronidazole	1 (8.3)
Surgery	8	Azithromycin	1 (12.5)
		Cephalexin	6 (75)
		Ciprofloxacin	1 (12.5)

## Discussion

This is the first point prevalence survey conducted in our hospital. It serves as baseline data to identify areas for strengthening the antimicrobial stewardship program. The overall prevalence of antimicrobial use in our study was 54.7%. The point prevalence of antimicrobial use across different studies in India varies widely, from 81.4% to 21.2% (Table 8). This difference may be attributed to variation in patient characteristics and disease burden. Differences in diagnostic facilities and prescribing practices may also contribute. 

**Table 8:** Comparative analysis of prevalence of antimicrobial use in various point prevalence surveys conducted in India.

Author & year of publication	Number of patients admitted in wards	Number of patients on antimicrobials	Point prevalence of antimicrobial use
Agrawal et al., 2025 [11]	215	175	81.4%
Borah et al., 2024 [12]	1396	1109	79.4%
Kumar et al., 2023 [13]	864	682	78.9%
Purohit, 2024 [14]	470	360	76.6%
Chadalavada et al., 2021 [15]	945	645	68.2%
Vadivoo et al., 2019 [16]	502	325	64.7%
Bhattacharjee et al., 2025 [17]	3974	2369	59.6%
Singh et al., 2019 [18]	1750	1005	57.4%
Panditrao et al., 2021 [19]	3473	1747	50.3%
Nirula et al., 2022 [20]	217	101	46.5%
Mittal et al., 2023 [21]	802	299	37.3%
Najmi et al., 2019 [22]	241	77	31.9%
Chakraverty and Samanta, 2021 [23]	340	72	21.2%
Present study, 2026	351	192	54.7%

Our study reported a higher prevalence of antimicrobial use (54.7%) than studies in China (28.2%) [24], Japan (29.2-33.5%) [25], Italy (40%) [26], Latin America (47.9%) [27], and Turkey (48.9%) [28]. However, the prevalence in our study is lower than the pooled point prevalence reported in a meta-analysis in Africa (64.2%) [29]. High antimicrobial use observed in this study may reflect empirical prescribing practices; however, a causal relationship cannot be established. 

The finding of predominant use of third-generation cephalosporin aligns with several other studies in India (Table 9). Similar to our study, the ‘Watch’ group of antimicrobials was prescribed more often than the ‘Access’ group in several other studies conducted in India (Table 10). This pattern deviates from the recommendation of the WHO, which advocates for ≥70% ‘Access’ group antimicrobial use [30]. The high reliance on 'Watch' antimicrobials, particularly third-generation cephalosporins, suggests empirical, broad-spectrum prescribing, possibly due to diagnostic uncertainty, a lack of institutional antimicrobial guidelines, and clinical preference. The overuse of 'Watch' agents may potentiate the acceleration of antimicrobial resistance. Overall, this finding highlights a critical need to realign prescribing practices with global recommendations. Increasing the proportion of 'Access' group use, whenever clinically appropriate, may be considered as a priority to support antimicrobial stewardship efforts. The absence of 'Reserve' antimicrobial use while seemingly positive may also reflect limited availability, and absence of confirmed multidrug-resistant infections, rather than optimal stewardship, warranting further evaluation. 

**Table 9:** Comparative analysis of the most common antimicrobial agents prescribed across different studies in India

Author & year of publication	Most common antimicrobial agents		
	1st	2nd	3rd
Agrawal et al., 2025 [11]	Ceftriaxone	Metronidazole	Amoxicillin clavulanic acid
Borah et al., 2024 [12]	Ceftriaxone	Piperacillin	Metronidazole
Purohit, 2024 [14]	Ceftriaxone	Amoxicillin clavulanic acid	Piperacillin tazobactam
Chadalavada et al., 2021 [15]	Ceftriaxone	Levofloxacin	Meropenem
Vadivoo et al., 2019 [16]	Cefotaxime	Penicillin	Metronidazole
Bhattacharjee et al., 2025 [17]	Ceftriaxone	Metronidazole	Amikacin
Singh et al., 2019 [18]	Penicillin with beta-lactamase inhibitors	Cefuroxime	Ceftriaxone
Panditrao et al., 2021 [19]	Third-generation cephalosporins	Imidazole	Beta-lactam/beta-lactamase inhibitors
Nirula et al., 2022 [20]	Ceftriaxone	Amoxicillin clavulanic acid	Metronidazole
Mittal et al., 2023 [21]	Penicillin with beta-lactamase inhibitors	Third-generation cephalosporins	Aminoglycosides
Najmi et al., 2019 [22]	Penicillin with beta-lactamase inhibitors	Ceftriaxone	Ciprofloxacin, levofloxacin
Present study, 2026	Cefotaxime	Ceftriaxone	Metronidazole

**Table 10:** Comparative analysis of antimicrobial use as per the WHO AWaRe classification in different studies conducted in India AWaRe: Access, Watch, and Reserve

Author & year of publication	Access	Watch	Reserve
Borah et al., 2024 [12]	28.8%	69.5%	1.7%
Panditrao et al., 2021 [19]	38%	57.9%	4.1%
Bhattacharjee et al., 2025 [17]	32.7%	57%	5.1%
Purohit, 2024 [14]	31.8%	54.5%	13.6%
Kumar et al., 2023 [13]	42.4%	53%	5.5%
Agrawal et al., 2025 [11]	47%	51.8%	1.2%
Present study, 2026	35.1%	64.9%	0

Like our study, the predominant use of parenteral antimicrobials was also reported in a multicentric point prevalence survey conducted in India (86.5%) covering 9652 patients [31]. It may be due to the severity of illness, lack of appropriate switch from parenteral to oral, or the misconception that parenteral antimicrobials work better than oral antimicrobials. Prolonged use of intravenous antimicrobials can lead to prolonged length of hospital stay, increased healthcare costs, and increased risk of catheter-related complications. Antimicrobial stewardship guidelines recommend antimicrobial review within 48-72 hours and switch to oral therapy whenever clinically appropriate [32].

A high proportion of empirical therapy, like our study, is documented in several other studies in India [12,13]. Limited antimicrobial susceptibility test-guided therapy suggests a lack of targeted therapy and de-escalation necessary for reducing broad-spectrum antimicrobial exposure. Similar to our study, quality indicators of antimicrobial use, such as documentation of stop date and mentioning of reason for antimicrobial use, are poor in several other studies in India [15,18,21]. 

The findings of this study have important implications for antimicrobial stewardship. Priority interventions include strengthening microbiological diagnostics, promotion of culture-based therapy, documentation of antimicrobial indication and stop dates, promoting intravenous to oral switch, and developing a hospital antimicrobial policy aligned with AWaRe recommendations. 

Limitations of the study are that it provides a snapshot of antimicrobial use at a single point in time, only in a single tertiary care centre. It cannot account for changes in antimicrobial use with seasonal variations. As data collection was performed across different wards on separate days, temporal variation may have introduced variability in prevalence estimates across wards. As the survey depended on medical record documentation, which may be incomplete, a comprehensive evaluation of prescribing appropriateness was not possible. Incomplete documentation in case records may have affected the accuracy of certain quality indicators. The study did not include inferential statistical analysis, limiting the ability to assess associations between variables. Despite these limitations, the use of the WHO-recommended methodology improves the validity of the study. The consistent pattern of high 'Watch' group antimicrobial use and excessive intravenous prescribing observed in this study aligns with the existing literature, supporting the robustness of the study.

## Conclusions

This study demonstrates suboptimal adherence to WHO AWaRe recommendations, with a predominance of 'Watch' antimicrobials and high empirical and parenteral use. There is a need for strengthening the antimicrobial stewardship programs in the healthcare facility with the following priorities: Documentation of indication to start antimicrobial, promotion of targeted therapy, increasing prescribing of 'Access' group antimicrobials, encouraging use of oral antimicrobial, and stop date documentation. Conducting point prevalence surveys regularly, followed by structured feedback to clinicians, may contribute to optimizing antimicrobial use and supporting stewardship efforts within similar healthcare settings. 
